# AdipoRon Mitigates Anxiety and Depression Symptoms in Chronic Sleep‐Restricted Mice via Modulation of Neuroinflammatory Responses

**DOI:** 10.1111/jcmm.70770

**Published:** 2025-08-05

**Authors:** Beriwan Malaei, Fereshteh Farajdokht, Gisou Mohaddes, Behzad Mansouri, Nasrin Hamidi, Mohammad Reza Alipour

**Affiliations:** ^1^ Neurosciences Research Center Tabriz University of Medical Sciences Tabriz Iran; ^2^ Department of Physiology, Faculty of Medicine Tabriz University of Medical Sciences Tabriz Iran; ^3^ Department of Biomedical Education California Health Sciences University, College of Osteopathic Medicine Clovis California USA; ^4^ Stem Cell Research Center Tabriz University of Medical Sciences Tabriz Iran

**Keywords:** AdipoRon, anxiety, depression, microglia, neuroinflammation, sleep

## Abstract

The contemporary lifestyle has resulted in an epidemic of chronic sleep restriction (SR), which has emerged as a substantial contributor to mood disorders, particularly anxiety and depression. This study examined the effectiveness of AdipoRon (Adipo), a synthetic adiponectin agonist, in mitigating anxious behaviours and depression‐like symptoms in a chronic SR mice model. Forty male Swiss albino mice were divided into four groups: wide platform (WP), SR, SR + melatonin (Mel), and SR + Adipo. Over 21 days, the animals underwent a modified multiple‐platform technique to induce SR, followed by daily intranasal treatments. The open field test and elevated plus maze were conducted to assess anxiety‐related behaviours, while the forced swimming test and sucrose splash test were used to assess depressive symptoms. Serum corticosterone concentrations, a marker of microglia activation, and pro‐inflammatory mediators (NF‐кB and IL‐1β) in the prefrontal cortex (PFC) were also examined. Intranasal administration of Adipo markedly decreased anxiety‐like behaviours, despair, and apathy in sleep‐restricted mice compared to the SR group. Adipo therapy also reduced pro‐inflammatory proteins and ionised calcium‐binding adaptor molecule‐1 (Iba‐1)‐positive cells in the PFC and restored serum corticosterone levels. These outcomes emphasise the therapeutic benefit of Adipo as an alternative therapy for mood disorders associated with SR.

## 1 Introduction

Sleep is a naturally occurring condition of altered consciousness marked by diminished awareness of and engagement with the external environment. For optimal functioning, persons aged 18–64 need 7–9 h of sleep every night, while those 65 and older need 7–8 h [1]. Sleep serves as an essential component for maintaining overall health and well‐being, as it affects a chain of physiological events in both peripheral and central tissues. In peripheral tissues, sleep supports metabolic homeostasis, physical healing and restoration, hormone balance, and immunologic function. In central tissues, sleep is necessary for neurogenesis, synaptic plasticity, cognitive processes, clearance of metabolic waste such as beta‐amyloid, and the regulation of neurotransmitter systems that are critical for mental health and emotional stability [2].

The quality and duration of sleep have been increasingly compromised in modern life, leading to a widespread sleep loss epidemic that impacts millions of people globally [3, 4]. Various factors, including irregular work hours, long‐term lifestyle pressures, and the widespread influence of technology, combine drastically, disrupting sleep patterns and lowering their duration and quality [4].

Sleep restriction (SR), defined as sleep ≤ 6 h per night, adversely affects the neurological system, resulting in decreased neurogenesis and neuroplasticity, diminished cognitive functions, immune system disruptions, disruption of the blood–brain barrier (BBB), and disturbed emotional regulation [5, 6].

Chronic SR particularly impacts the prefrontal cortex (PFC), a key brain area responsible for emotion processing, leading to heightened emotional reactivity and diminished stress management capabilities, thereby increasing liability to mental health problems, notably anxiety and depression [7, 8]. Additionally, sleep and mood are mutually reinforcing, as sleep loss can initiate anxiety and depression, which can exacerbate sleep issues, thereby creating a vicious cycle [9].

The aetiology of psychological disorders induced by SR encompasses multiple interrelated processes, including diminished serotonergic neurotransmission, circadian rhythm disruption, hyperactivity of the hypothalamic–pituitary–adrenal (HPA) axis, and neuroinflammation [5, 10]. A key component of the body's stress response system, the HPA axis, is controlled by the circadian rhythm, which causes cortisol levels to rise in the morning and fall in the daytime [11]. Nevertheless, chronic SR disrupts the normal diurnal rhythm of cortisol, often causing a phase shift that leads to a biphasic pattern with peaks in the morning and evening [12], resembling the cortisol circadian rhythm observed in anxiety and depression [13].

Chronic SR‐evoked persistent activation of the HPA axis triggers a cascade of events that promote the activation of the nuclear factor (NF)‐κB pathway and the release of pro‐inflammatory cytokines along with microglial activation [5, 7]. This process contributes to chronic low‐grade neuroinflammation, resulting in diminished monoamine neurotransmitters and neurotrophic factors while increasing oxidative stress. These neurobiological alterations disrupt the normal functioning of neural networks implicated in mood regulation and stress response, ultimately resulting in depressive symptoms and emotional dysregulation [5, 14, 15].

The pineal gland produces the hormone melatonin (Mel), fundamental to establishing the sleep–wake cycle, circadian rhythms and synchronising the internal biological clock with the external environment. Although Mel may enhance sleep quality and alleviate depressive symptoms in some cases, its efficacy might differ among individuals, and the effects are often moderate [16]. The increasing prevalence of SR in modern society and its detrimental impacts on mental health, coupled with the limited efficacy of available treatment options for sleep‐associated mood disorders, highlight the need for innovative and more focused approaches.

Multiple studies have established a negative association between circulating adiponectin levels and depression severity, implying that individuals with depression exhibit markedly decreased adiponectin levels. Animal studies have shown that adiponectin deficiency enhances vulnerability to depression‐like behaviours, whereas exogenous adiponectin administration provides antidepressant‐like benefits [17, 18]. Consequently, enhancing the activity of the central adiponergic system may provide a potential avenue for creating new and multifaceted antidepressant therapies. AdipoRon (Adipo) is a synthetic adiponectin agonist that interacts with and activates both AdipoR1 and AdipoR2 receptors, thereby mimicking the effect of adiponectin [19]. This agonist has shown promise in the management of different neurological conditions, primarily by its diverse effects, involving the promotion of neurogenesis and serotonergic neurotransmission, and anti‐inflammatory and antioxidant functions. In addition, Adipo has demonstrated great promise in addressing anxiety and depression in animal models, such as chronic stress models and a rat model of Parkinson's disease, mainly through the suppression of neuroinflammatory reactions, inhibiting the tryptophan/kynurenine pathway, increasing serotonin turnover, promoting hippocampal neurogenesis, and modulating the HPA axis [20, 21].

Despite the promising effects of Adipo on mood disorders, its efficacy in treating anxiety and depression induced by SR remains unexplored. Therefore, this research aimed to explore the effects of long‐term intranasal Adipo on anxious and depressive‐like behaviours in a mouse model of chronic SR and to assess its influence on inflammatory factors in the PFC and the HPA axis function.

## 2 Material and Methods

### 2.1 Animals

Forty male Swiss albino mice, around 3 months old and weighing between 25 and 30 g, were acquired from the animal facility at Tehran University of Medical Sciences, Iran. The animals were accommodated in normal polycarbonate cages, each containing five mice, under conventional laboratory settings: ambient temperature set at 22°C ± 2°C, humidity adjusted at 55% ± 5%, and a 12‐h light/dark period (lights on at 07:00 AM) Throughout the experiment, mice were provided with filtered drinking water and standard rodent chow on an ad libitum basis. One week before the start of the experimental procedures, the animals were given the opportunity to adjust to the research facility settings. All experimental procedures adhered to the Instructions for the handling and utilisation of research animals established by the National Institutes of Health (NIH Publication No. 85‐23, revised 1996) and received approval from the Regional Ethics Committee of Tabriz University of Medical Sciences (Approval No. IR.TBZMED.AEC.1402.029). All measures were implemented to lessen animal pain and decrease the quantity of animals utilised.

### 2.2 Experimental Design

**FIGURE 1:**
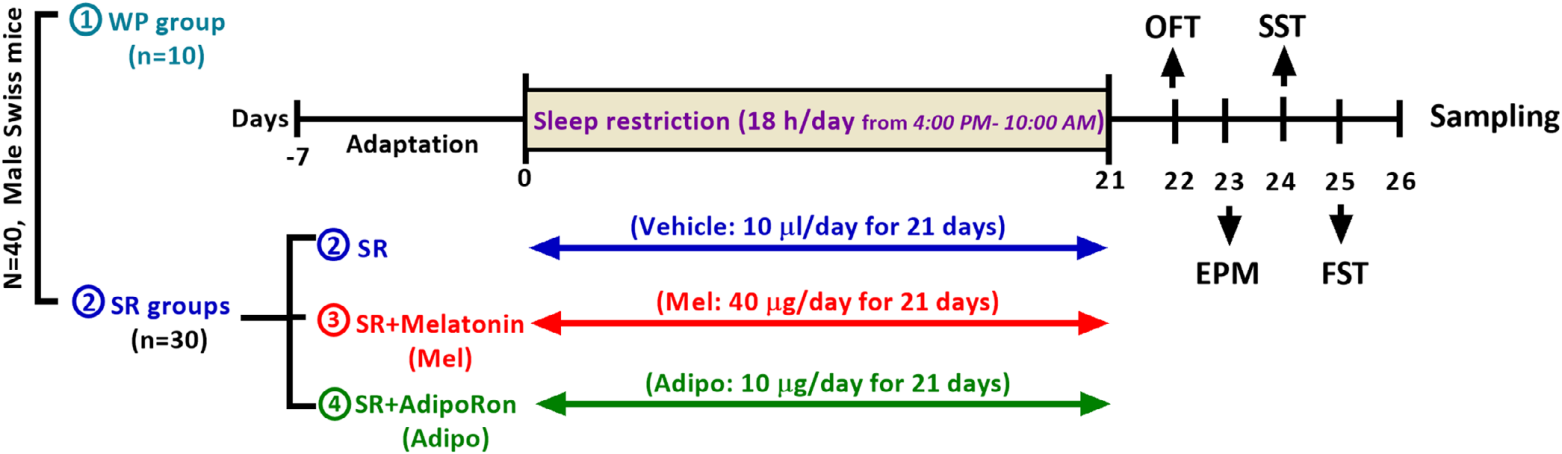
Experimental design. (WP, wide platform; SR, sleep restriction group; Mel, melatonin; Adipo, AdipoRon; EPM, elevated plus maze; FST, forced swimming test; OFT, open field test; SST, sucrose splash test).

Randomly, 10 mice were put into each of the four experimental groups: the wide platform control group (WP), SR, SR + melatonin (Mel), and SR + Adipo (Adipo). All treatments were prepared using 1% dimethyl sulfoxide (DMSO) in saline as the vehicle and administered intranasally daily for 21 days, between 2:00 and 4:00 PM, prior to subjecting the SR protocol. The WP and SR groups received 10 μL/mouse of the vehicle, while the Mel group was administered Mel (40 μg/mouse in 10 μL), and the Adipo group received Adipo (10 μg/mouse in 10 μL) [21]. The dosage of Adipo was selected based on the findings from our previous dose–response investigation, which indicated that this dosage (10 μg) produced the most significant anxiolytic and antidepressant effects in similar behavioural paradigms [21]. The intranasal route was chosen to minimise systemic exposure and ensure direct access to the brain, thereby bypassing the BBB. Besides, this method is cost‐effective, enabling effective central nervous system (CNS) concentrations with reduced doses of costly compounds. The schedule and order of the interventions carried out in this experiment are depicted in Figure 1.

### 2.3 SR Procedure

A Plexiglas tank measuring 42 × 30 × 20 cm was filled with water up to 1 cm below the top of 12 circular platforms measuring 1.5 cm in diameter. The animals could traverse the tank and move between platforms freely. The animals were subsequently positioned within the tanks daily from 4:00 PM to 10:00 AM for 21 successive days. The animals were permitted to recover and sleep in their home cages for 6 h from 10:00 AM to 4:00 PM. The WP control group was kept in similar tanks with wider platforms (6 cm in diameter), which enabled them to sleep normally with no risk of slipping into the water. From the cage cover, food pellets were suspended, and water was accessible within the tank [22].

### 2.4 Behavioural Assessments

All behavioural assessments were done in a quiet room from 9:00 AM to 2:00 PM. Before the test, every animal was familiarised with the testing room for 30 min. All sessions were captured with a ceiling‐mounted camera and processed with EthoVision software (Noldus Information Technology, Netherlands). The experimenter was blinded to treatment groups during testing and analysis. To minimise smell cues, all equipment was cleaned with 70% ethanol between sessions.

#### 2.4.1 Open Field Test (OFT)

The test was performed within a square field (50 × 50 × 40 cm). After positioning each mouse in the centre, marked by a black line, the animal was allowed to travel the device for 5 min while activities were tracked using a video tracking system. A blinded investigator analysed different activities, including grooming time, rearing episodes, entries into the central region, and centre time spent [23].

#### 2.4.2 Elevated Plus Maze (EPM)

A maze was made up of two arms with open tops (30 × 5 × 0.5 cm) and two arms with closed tops (30 × 5 × 15 cm), elevated 50 cm from the ground. Individual mice were positioned at the arena's centre, facing an open arm, and permitted to investigate the maze for 5 min. We recorded behavioural variables such as the amount of time spent in the open arms and the number of entrances into the open arms [23].

#### 2.4.3 Forced Swimming Test (FST)

For this test, a transparent cylindrical container of 20 cm in diameter and 40 cm in height was filled with 30 cm of tap water at a temperature of 25°C ± 2°C. A six‐minute swimming session was conducted on mice, and the final 4 min of immobility were recorded. Immobility was referred to as staying immobile in the water with little activity to sustain the head above water. Mice were dried with a towel and then allowed to rest under a lamp for 30 min following the test. The water was replaced following every swim [23].

#### 2.4.4 Sucrose Splash Test

Apathy‐like behaviours and motivational deficits were evaluated using the sucrose splash test, which was chosen due to its ease of use, speed, and proven validity as a measure of motivational and self‐care deficits linked to depression‐like states in rodents. In addition, compared to the sucrose preference test, the sucrose splash test does not require prolonged habituation or deprivation periods or multi‐day testing, indicating fewer stresses and practical difficulties. For this purpose, each animal was housed in transparent Plexiglas cages and permitted to habituate to the container for 10 min. After that, the back was sprayed with a 10% sucrose solution, and the duration of grooming the face and body was recorded for 5 min [24]. Decreased grooming time was interpreted as indicative of diminished motivation and self‐care, reflecting apathy‐like behaviour in rodents.

### 2.5 Sampling

Following the behavioural assessments, the animals underwent anaesthesia and were decapitated for brain tissue harvesting. Upon collecting blood from the heart, the samples were left to clot at room temperature for 20 min. The serum was then extracted by centrifugation at 4000 rpm for 10 min at 4°C. For molecular analysis, the PFC was cut out from half of the animals and then frozen in liquid nitrogen. The animals in the other half were transcardially perfused with a cold 4% paraformaldehyde solution, and the entire brain tissue was removed for further histological examination. To reduce fluctuation in corticosterone levels caused by diurnal oscillations, all samples were obtained between 10:00 AM to12:00 PM.

### 2.6 Serum Corticosterone Levels

Blood serum was examined for corticosterone using an enzyme‐linked immunosorbent assay (ELISA) kit (Elabscience, China), according to the directions supplied by the company. The sensitivity of the test is 1.93 ng/mL, and the detection range is 3.12–200 ng/mL.

### 2.7 Western Blotting

Pro‐inflammatory cytokine expression levels, notably NF‐κB and IL‐1β, were quantified in PFC tissue samples using a previously established method [25]. Briefly, a protease inhibitor cocktail was added to the RIPA lysis solution and used to homogenise PFC tissue samples. Thereafter, the homogenate underwent centrifuging at 12,000×*g* for 15 min at 4°C, and the emerged supernatant was harvested, with total protein content quantified employing the Bradford Protein Quantification kit (DB0017, DNAbioTech, Iran). Protein separation was accomplished via SDS‐PAGE. The isolated proteins were subsequently electrophoretically transmitted to a polyvinylidene difluoride (PVDF) membrane (Bio‐Rad Laboratories, CA, USA). After blocking, primary antibodies against NF‐κB p65 (Cat No: ab16502, Abcam), NF‐κB p65 phospho S536 (Cat No: ab76302, Abcam) and IL‐1β (Cat No. ab254360, Abcam) at a dilution of 1:1000, and β‐actin (Cat No: ab8227, Abcam) at a dilution of 1:2500 were applied to the membranes and incubated overnight. Following three consecutive 10‐min PBS washes, a secondary antibody, goat anti‐rabbit IgG H&L (HRP) (Cat No: ab6721; Abcam), was applied to the membranes and left to incubate at room temperature for 2 h. The immunoreactive bands were subsequently visualised using an enhanced chemiluminescence (ECL) detection reagent and exposed to X‐ray film. The resultant strings of protein were captured digitally and processed with ImageJ 1.62 computer software.

### 2.8 Immunofluorescence Staining

The fixed brain tissues underwent dehydration in a 30% sucrose solution for 48 h. Coronal slices (10 μm) were prepared with a cryostat at −25°C and subsequently mounted on poly‐lysine‐coated glass slides. Sections were selected from the PFC between bregma +2.0 mm and +1.5 mm, and treated with 10% regular serum or 1% BSA in TBS at room temperature for 2 h to decrease unspecific binding. The slides were immersed in a rabbit anti‐ionised calcium‐binding adapter molecule 1 (Iba‐1) primary antibody diluted in 1% BSA overnight at 4°C. After three washes with PBS, sections were treated with FITC‐conjugated goat anti‐rabbit IgG secondary antibodies (1:400) and DAPI (1:1000) for nuclear counterstaining for an hour at ambient temperature. Afterwards, the slides were coated with a fluorescence mounting reagent. Three non‐overlapping fields were chosen at random from each section for imaging using an Olympus BX50 fluorescence microscope with appropriate filter sets for DAPI and Alexa Fluor dyes at a magnification of 200×. Iba‐1‐positive cells were manually counted by an investigator unaware of group assignments. The mean number of Iba‐1‐positive cells per field was determined for each animal, and the group averages were employed for statistical analysis.

### 2.9 Data Analysis

Analysis of statistics was accomplished using GraphPad Prism software version 9. The results are displayed as the mean plus or minus the standard error of the mean (SEM). One‐way analysis of variance (ANOVA) was employed to evaluate statistical disparities in normally distributed data, followed by the Tukey post hoc test. The criterion for determining statistical significance was set at *p* < 0.05.

## 3 Results

### 3.1 Intranasal Adipo Alleviated Anxious Behaviours in Chronic SR‐Subjected Mice

#### 3.1.1 Open Field Test (OFT)

**FIGURE 2:**
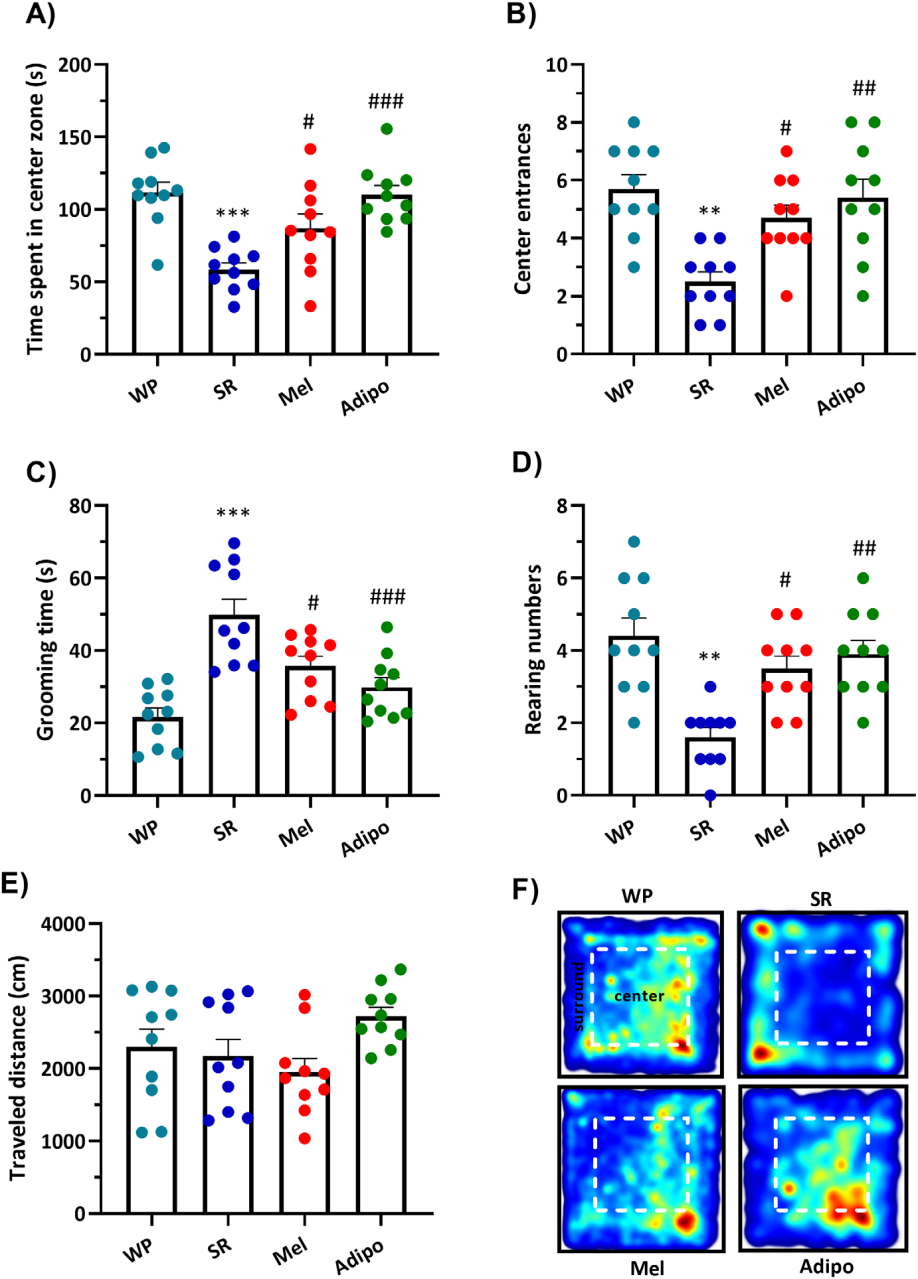
Effects of intranasal AdipoRon (Adipo) and melatonin (Mel) on anxiety‐like behaviours in chronic sleep‐restricted mice. (A) Cumulative time in the central zone, (B) center entrances, (C) grooming time, (D) rearing numbers and (E) travelled distance in the open field test (OFT). (F) Representative heat map of the animal activity in the OFT. Statistics are reported as mean ± SEM (*n* = 10/group). Statistical significance: **p < 0.01 and ***p < 0.001 compared to the WP group; #*p* < 0.05, ##*p* < 0.01, ###*p* < 0.001 compared to the SR group. (WP, wide platform; SR, sleep restriction group).

The findings of the one‐way ANOVA indicated substantial differences in the time spent in the center zone (*F*_(3,36)_ = 11.80, *p* < 0.001), center entrances (*F*_(3,36)_ = 8.635, *p* = 0.0002), grooming time (*F*_(3,36)_ = 14.12, *p* < 0.0001), rearing numbers (*F*_(3,36)_ = 10.32, *p* < 0.0001) among the experimental groups in the open field test. However, no significant change was observed in the travelled distance (*F*_(3,36)_ = 2.516, *p* = 0.0737) among the groups. Multiple comparisons showed that the sleep‐restricted mice spent a shorter time in the center zone (Figure 2A, *p* < 0.001), while they showed longer grooming time (Figure 2C, *p* < 0.001) than the WP group. Moreover, the number of center entrances (Figure 2B, *p* < 0.01) and rearing (Figure 2D, *p* < 0.01) in the SR group was lower than those of the WP group.

Conversely, intranasal delivery of either Adipo or Mel resulted in significant increases in time spent in the center (*p* < 0.05 for Mel and *p* < 0.001 for Adipo), center entrances (*p* < 0.05 for Mel and *p* < 0.01 for Adipo) and rearing numbers (*p* < 0.05 for Mel and *p* < 0.001 for Adipo) compared to the untreated SR group. Besides, both intranasal administration of Adipo (*p* < 0.001) and Mel (*p* < 0.05) considerably increased grooming time in SR‐subjected mice. Nonetheless, no notable disparity was observed among the groups in the travelled distance (Figure 2E).

#### 3.1.2 Elevated Plus Maze (EPM)

**FIGURE 3:**
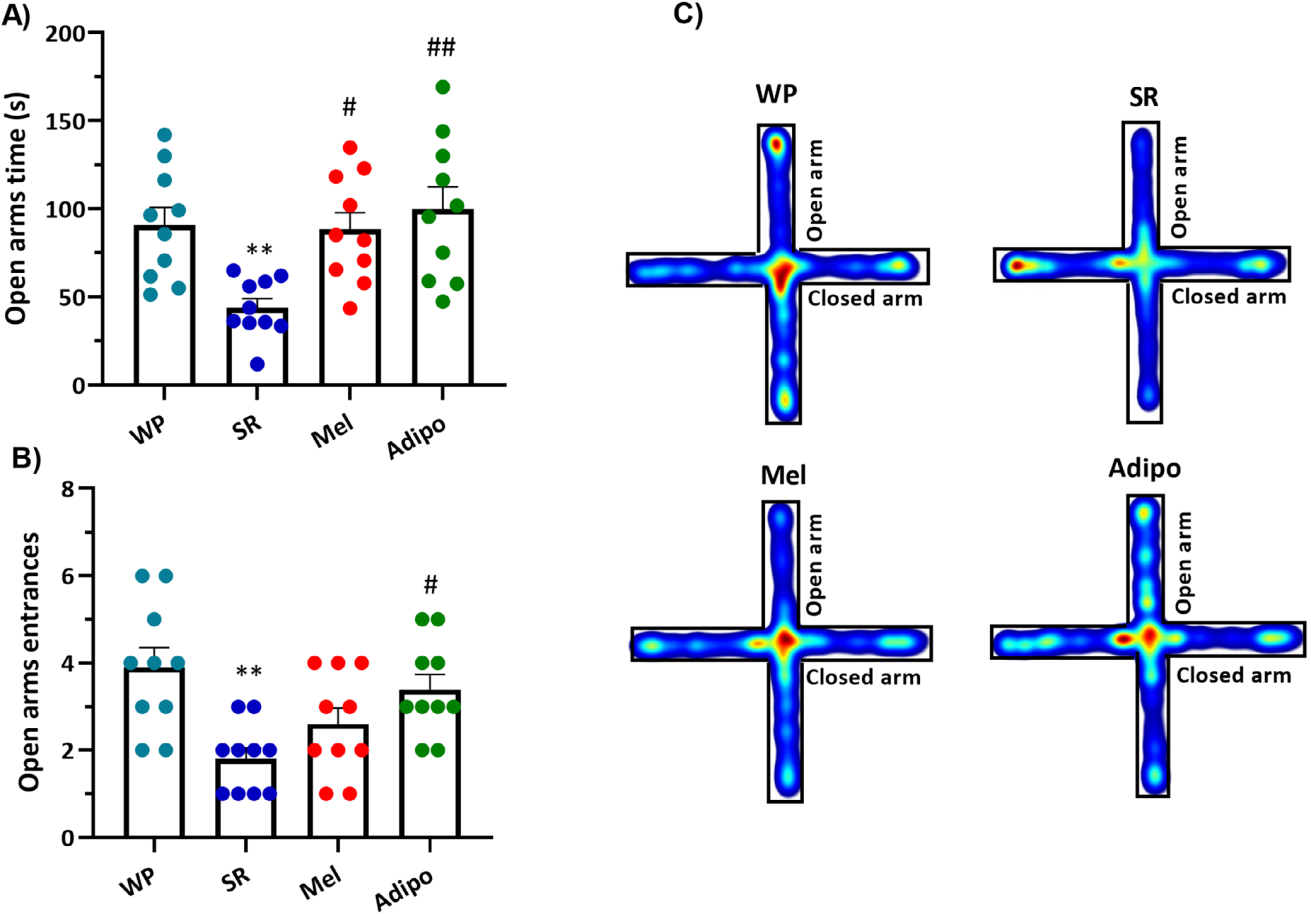
Impacts of intranasal AdipoRon (Adipo) and melatonin (Mel) on anxious behaviours in sleep‐restricted mice. The elevated plus maze (EPM) test was employed to evaluate (A) Open arm time and (B) frequency of open arm entries across different treatment groups. Statistics are shown as mean ± SEM (*n* = 10/group). (C) Heatmap representation of the animal activity in the maze over 5 min; red indicates the highest residence and blue the lowest. Statistical significance: **p < 0.01 compared to the WP group; #*p* < 0.05 and ##*p* < 0.01 compared to the SR group. (WP, wide platform; SR, sleep restriction group).

We also found significant differences in two key parameters of the EPM test, including time spent in the open arms (*F*_(3,36)_ = 6.519, *p* = 0.0012) and open arms entries (*F*_(3,36)_ = 6.463, *p* = 0.0013) among the study groups. Inter‐group comparisons showed that the SR group spent less time (Figure 3A, *p* < 0.01) in the open arms and had a lower number of entrances to the open arms (Figure 3B, *p* < 0.01) compared to the WP group. Nevertheless, intranasal administration of Adipo markedly resulted in a significant increase in both open arms time (*p* < 0.01) and open arm entries (*p* < 0.05) compared to the SR group. Besides, Mel administration significantly increased the time spent in the open arms (*p* < 0.05) in sleep‐restricted mice. However, it did not elicit a statistically substantial effect on the frequency of open arms entries.

### 3.2 Intranasal Treatment of Adipo Attenuated Depressive‐Like Symptoms in Chronic SR‐Subjected Mice

**FIGURE 4:**
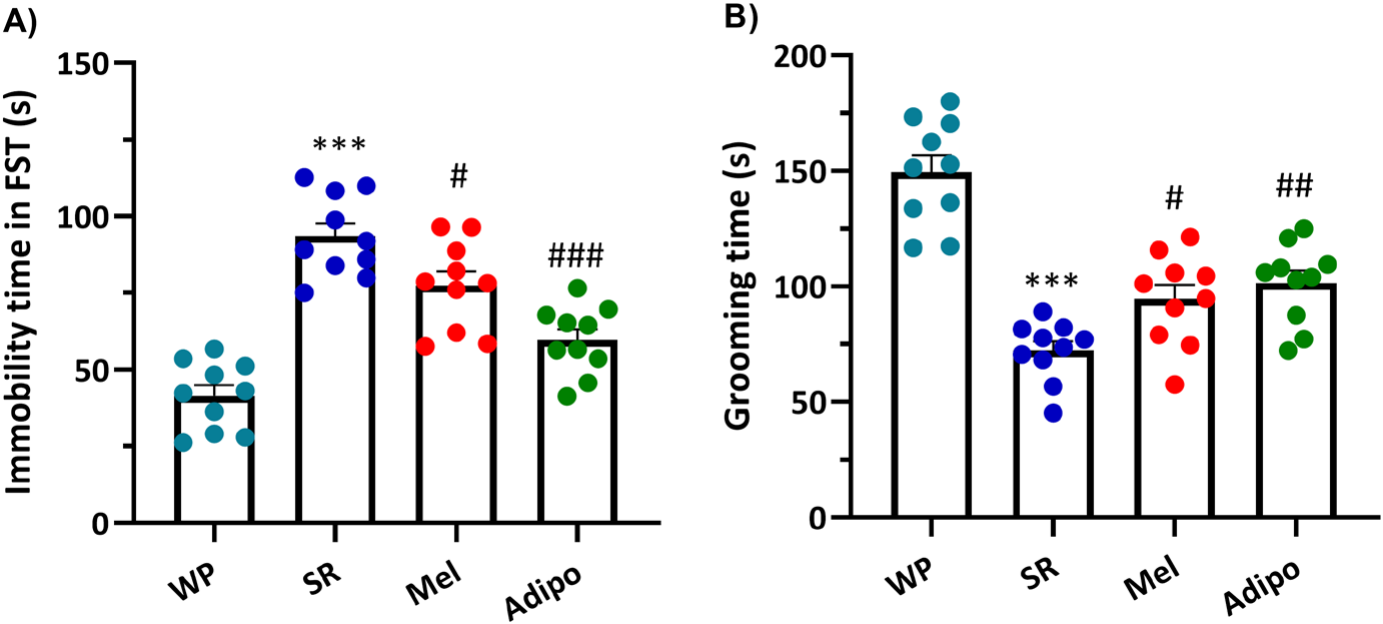
The effect of intranasal AdipoRon (Adipo) and melatonin (Mel) on depressive‐like behaviours in the study groups. (A) Immobility time in the forced swim test (FST). (B) Grooming time in the sucrose splash test. The results are reported as mean ± SEM (*n* = 10/group). Statistical significance: ***p < 0.001 compared to the WP group; #*p* < 0.05, ##*p* < 0.01, ###*p* < 0.001 compared to the SR group. (WP, wide platform; SR, sleep restriction group).

The results of one‐way ANOVA of immobility time (*F*_(3,36)_ = 32.03, *p* < 0.0001) in the FST and grooming time in the sucrose splash test (*F*_(3,36)_ = 30.88, *p* < 0.001) indicated significant differences across the groups. Post hoc analysis confirmed that chronic SR exposure obviously prolonged the duration of immobility in the FST (Figure 4A , *p* < 0.001), while the duration of grooming was reduced in the sucrose splash test (Figure 4B, *p* < 0.001) relative to the WP group. Nevertheless, intranasal treatments either with Adipo (*p* < 0.001 for immobility and *p* < 0.01 for grooming time) or Mel (*p* < 0.05 for both) significantly decreased immobility time and improved grooming time in sleep‐restricted mice.

### 3.3 Adipo Treatment Decreased Serum Corticosterone Levels in Sleep‐Restricted Mice

**FIGURE 5:**
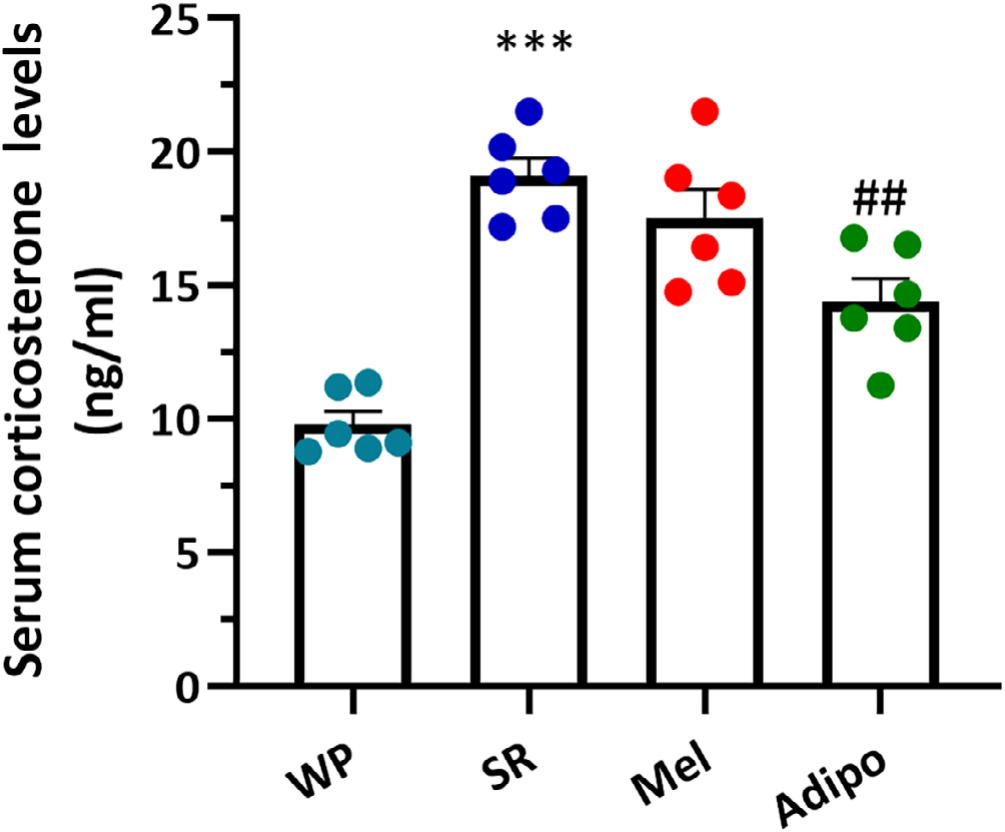
Effects of intranasal AdipoRon (Adipo) and melatonin (Mel) on serum corticosterone levels in sleep‐restricted mice. Data are represented as mean ± SEM (*n* = 6). ***p < 0.001 compared to the WP group; ##*p* < 0.01 compared to the SR group. (WP, wide platform; SR, sleep restriction group).

The serum corticosterone levels were checked to investigate the stress hormone‐related reaction, and a substantial disparity was observed between the groups (*F*_(3,20)_ = 26.91, *p* < 0.0001). Multiple comparisons revealed that chronic SR resulted in a substantial rise in serum corticosterone levels in comparison to the WP group (Figure 5, *p* < 0.001). Nevertheless, treatment with Adipo significantly attenuated this increase in serum corticosterone levels (*p* < 0.01), suggesting a protective effect against SR‐induced hormonal changes. In contrast, the Mel group did not differ significantly from the SR group regarding serum corticosterone levels.

### 3.4 Intranasal Adipo Therapy Suppressed Microgliosis in the PFC of Sleep‐Restricted Mice

**FIGURE 6:**
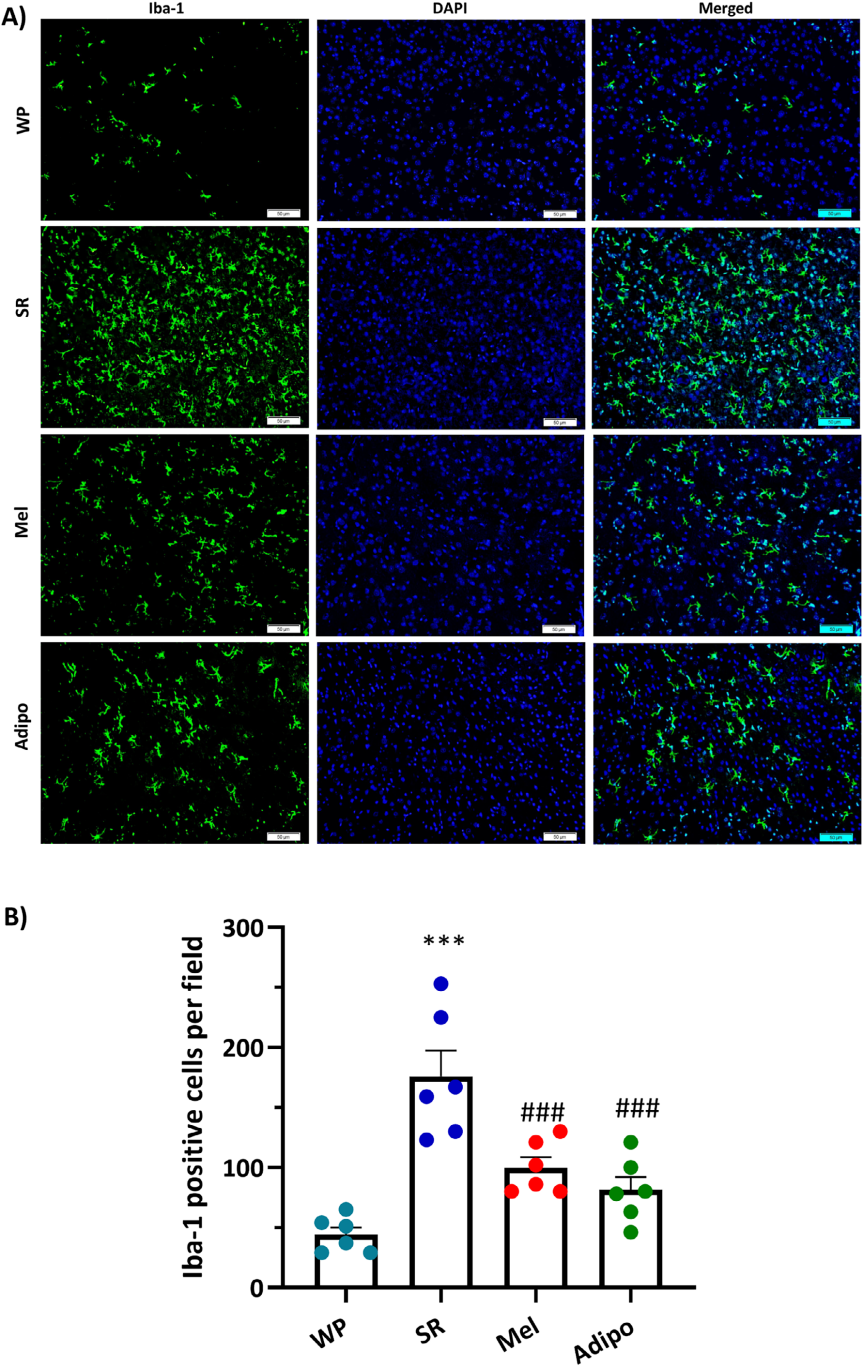
Effects of intranasal AdipoRon (Adipo) and melatonin (Mel) on microglial activation in the prefrontal cortex (PFC) of sleep‐restricted mice. (A) Immunofluorescence staining of Iba‐1 (green) in the PFC of different groups. DAPI (Blue) was used to label the cell nucleus. Scale bar, 50 μm; original magnification 200×. (B) Evaluation of the quantity of PFC cells expressing Iba‐1 following 21 days of SR. Data are represented as mean ± SEM. ***p < 0.001 compared to the WP group; ###*p* < 0.001 compared to the SR group. (WP, wide platform; SR, sleep restriction).

The immunoreactivity of Iba‐1 (green) was evaluated in the PFC to ascertain the relationship between inflammation and microglial cell activation following chronic SR. Based on the results of one‐way ANOVA, a significant variation (*F*_(3,20)_ = 18.02, *p* < 0.001) was found across the experimental groups. Compared to the WP group, the sleep‐restricted mice exhibited greater numbers of Iba‐1‐positive cells (Figure 6, *p* < 0.001). Nonetheless, treatment with Mel or Adipo significantly reduced the quantity of Iba‐1+ cells in the PFC of SR‐exposed animals (*p* < 0.001 for both treatments).

### 3.5 Intranasal Adipo Treatment Suppressed Inflammatory Responses in the PFC After SR

**FIGURE 7:**
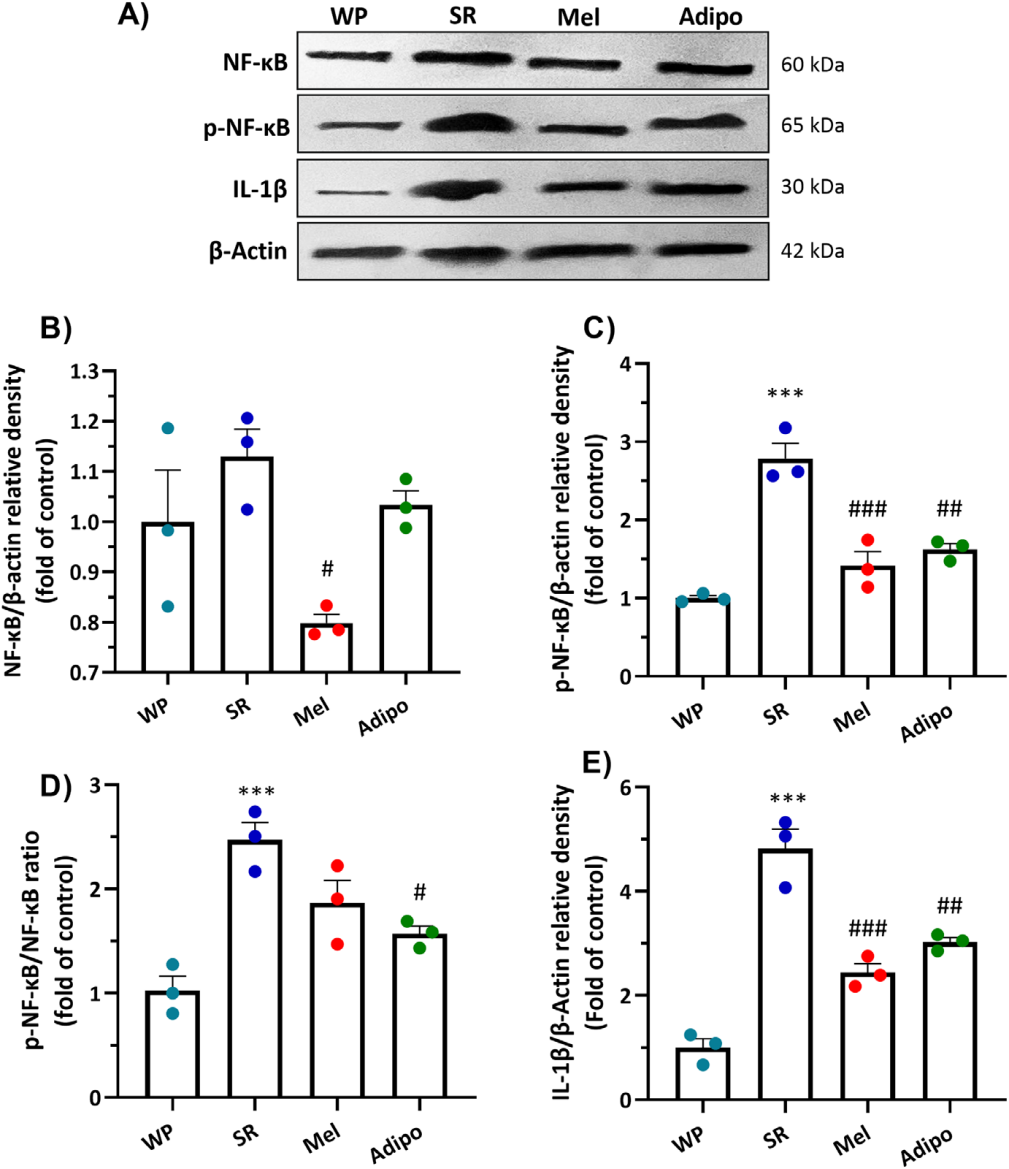
Impacts of intranasal AdipoRon (Adipo) and melatonin (Mel) treatments on inflammatory mediators, including (B) NF‐κB p65, (C) phospho (p)‐NF‐κB, (D) p‐NF‐κB/NF‐κB ratio, and (E) IL‐1β in the prefrontal cortex of sleep‐restricted mice. **(A)** Representative images of protein bands identified by immunoblotting. Data are represented as mean ± SEM (*n* = 3/group). ***p < 0.001 compared to the WP group; #*p* < 0.05, ##*p* < 0.01, ###*p* < 0.001 compared to the SR group. (WP, wide platform; SR, sleep restriction group).

One‐way ANOVA validated significant differences among the four groups in NF‐κB (*F*_(3,8)_ = 5.304, *p* = 0.026), p‐NF‐κB (*F*_(3,8)_ = 30.84, *p* < 0.001), and IL‐1β (*F*_(3,8)_ = 47.34, *p* < 0.001) protein expression in the PFC. As shown in Figure 7A–E, protein expressions of p‐NF‐κB (Figure 7C, *p* < 0.001), p‐NF‐κB/NF‐κB ratio (Figure 7D, *p* < 0.001), and IL‐1β (Figure 7E, *p* < 0.001) significantly increased in the PFC of SR‐subjected mice compared to the WP group. However, the Adipo group showed lower protein expression of p‐NF‐κB (*p* < 0.01), p‐NF‐κB/NF‐κB ratio (*p* < 0.05) and IL‐1β (*p* < 0.01) than the SR group. Moreover, Mel treatment significantly decreased protein levels of NF‐κB (Figure 7B, *p* < 0.05), p‐NF‐κB (*p* < 0.001) and IL‐1β (*p* < 0.001), while it had no significant effect on p‐NF‐κB/NF‐κB ratio in sleep‐restricted mice.

## 4 Discussion

The current research examined the efficacy of intranasal Adipo in alleviating depression and anxiety in a mouse model of chronic SR. The results demonstrated that Adipo significantly reduced anxiety‐like behaviours and diminished apathy‐like behaviours and despair in SR‐subjected mice. Furthermore, Adipo successfully decreased the heightened HPA axis activity, resulting in reduced chronic stress responses and improved mood regulation. Notably, Adipo suppressed neuroinflammation, a critical contributor to the onset of depression, by inhibiting the NF‐κB pathway and reducing microglial activation in the PFC. These findings underscore Adipo's promising role as a therapeutic agent in managing mood disorders linked to sleep disturbances.

A number of contemporary lifestyle factors contribute to chronic SR, such as heavy caffeine and alcohol usage, working night shifts, emotional stress, and overuse of electronic gadgets [26, 27]. These factors disrupt sleep patterns and significantly reduce sleep duration and quality, creating a vicious cycle that increases emotional distress and deteriorates mood regulation [28, 29]. At the same time, mental health issues disrupt sleep quality, resulting in heightened sleep fragmentation and abnormal sleep patterns [9, 29]. This interaction emphasises the pressing necessity for novel therapeutic alternatives to resolve the mental health issues arising from SR. In agreement with former reports [7, 30], we showed that chronic SR led to anxious behaviours, as evidenced by decreased time spent in the centre and fewer rearing numbers, along with increased grooming behaviour in the OFT. Additionally, chronic SR resulted in reduced time spent in the open arms and few entries into these areas in the EPM test. Furthermore, in the FST and sucrose splash test, chronic SR was associated with despair behaviour, motivational deficits, and self‐neglect, indicative of depressive symptoms. In contrast, intranasal administration of the adiponectin receptor agonist (Adipo) resulted in anxiety‐reducing effects and mitigated depression‐related symptoms in sleep‐restricted mice.

The mechanisms underlying anxiety and depression induced by chronic SR remain inadequately understood. According to preclinical and clinical evidence, the HPA axis activity and sleep loss are bidirectionally interrelated [12, 31]. Sleep loss or fragmentation acts as a substantial stressor that stimulates neuroendocrine stress systems and induces sustained activation of the HPA axis, which in turn promotes arousal and wakefulness, further interfering with normal sleep cycles [32]. On the other hand, HPA axis activation can cause fragmented or less deep sleep by raising cortisol release during times when it should be low, like at night. This loop intensifies stress responses and leads to several health complications, including psychological disorders and metabolic diseases [31]. Indeed, abnormalities in the HPA axis contribute to the development of anxiety and depression symptoms by disrupting monoamine neurotransmission, impairing synaptic plasticity, decreasing neurogenesis, and inducing neuroinflammation [33, 34]. Heightened HPA axis activity or disrupted circadian rhythms are frequently noted in individuals with depression. Therefore, addressing the aberrations of HPA axis activity is regarded as a promising approach for the development of novel antidepressant medications [35]. Similar to previous reports [36, 37, 38, 39], this study revealed high serum corticosterone levels along with anxious and depressive behaviours in SR mice. Conversely, Adipo therapy significantly decreased corticosterone levels and improved these symptoms in the SR‐exposed mice. In line with our findings, Azizifar et al. also showed that intranasal Adipo therapy decreased corticosterone serum concentration in hemiparkinsonian rats [21].

In addition to producing psychological distress, the abnormality in the HPA axis triggers an inflammatory reaction that involves glial cells activation, especially microglia, and the secretion of inflammatory compounds that promote neuroinflammation [40]. Accumulating preclinical [5, 7, 41] and clinical [42, 43] studies reported that acute or chronic sleep loss markedly increased pro‐inflammatory molecules while decreasing anti‐inflammatory proteins. There are a number of potential pathways that contribute to neuroinflammation in response to SR, including disturbances in circadian rhythms, hyperactivity of the HPA axis, and a compromised BBB that permits inflammatory factors from the periphery to enter the brain [7, 30]. Microglia, as immune‐regulating cells of the CNS, are especially sensitive to infections, long‐term stress, and irregular sleep patterns, all of which can raise the risk of neuropsychiatric conditions like depression [44]. Microglial cells are crucial for preserving CNS homeostasis by modulating sleep equilibrium, facilitating synaptic pruning, removing cellular debris, and participating in neuroinflammatory reactions [45]. Previous studies indicated that microglial activation, in conjunction with their release of cytokines and phagocytic activity, is influenced by the circadian clock. Notably, microglia show elevated levels of pro‐inflammatory cytokines during the light phase, while their highest phagocytic activity occurs during the dark phase [45]. Microglial activation is a fundamental element linking chronic sleep loss and the emergence of mood disorders [45, 46]. Sleep deprivation, whether temporary or long‐term, activates microglia, which in turn triggers NF‐κB and amplifies the production and secretion of pro‐inflammatory cytokines; these cytokines have been linked to mood disturbances [7, 47]. Conversely, the suppression of microglial activation may alleviate neuroinflammation and neurobehavioral abnormalities linked to sleep loss, including anxiety and depression [7, 47]. Besides, the interplay between the HPA axis dysregulation and neuroinflammation after sleep loss creates a vicious cycle that can damage neurotransmitter systems and disrupt neuronal circuits involved in processing emotions and responding to stress, particularly in the PFC, resulting in heightened anxiety and depressive symptoms [30, 41, 42]. In this study, we showed a significant increase in the number of Iba‐1^+^ cells in the PFC in SR mice, implying microglial activation. Moreover, we confirmed that the protein levels of p‐NF‐κB and IL‐1β were markedly increased after chronic SR. However, Adipo therapy effectively reversed all these changes in sleep‐restricted mice. Consistent with our findings, Liu et al. reported that sleep deprivation led to increased anxiety‐like behaviour accompanied by a higher number of Iba‐1^+^ microglial cells in the PFC. Notably, they found that treatment with minocycline, an anti‐inflammatory agent, effectively reduced both the anxiety‐like behaviours and microglial activation induced by sleep deprivation [7].

Adiponectin, recognised for its anti‐inflammatory properties, is crucial for mood regulation [48]. Multiple studies indicate that individuals experiencing depression generally exhibit reduced levels of adiponectin [49]. Moreover, lower circulating levels of adiponectin are accompanied by heightened vulnerability to mood disorders, whereas its administration has demonstrated antidepressant effects [17, 50]. Furthermore, long‐term exposure to stress, such as that experienced during SR, has been demonstrated to significantly reduce blood adiponectin levels, leading to behaviours resembling depression and dysregulation of the HPA axis, all of which were reversed with the administration of adiponectin [51]. Considering the established impacts of chronic SR on the stress system and heightened neuroinflammatory processes, it seems that augmenting adiponectin signalling through Adipo therapy could alleviate the emotional impairments linked to SR. Consistent with our findings, earlier research has proven that the stimulation of the adiponectin signalling pathway through the administration of Adipo yields anxiolytic and antidepressant effects in various rodent models, primarily by reducing neuroinflammation in brain regions linked to mood regulation, specifically the hippocampus and PFC [20, 21, 50].

Adipo primarily functions by activating adiponectin receptors, AdipoR1 and AdipoR2, which are extensively expressed in the CNS, particularly in regions associated with the control of emotions such as the PFC, amygdala, hippocampus, and hypothalamus [51, 52]. The expression patterns of these receptors are cell‐type specific; while AdipoR2 is present in both neuronal and glial populations, AdipoR1 is predominantly localised in neurons and is particularly abundant in cortical areas [53]. Notably, AdipoR1 is predominantly expressed in serotonergic neurons [48], and its activation has been linked to better mood regulation and reduced anxiety and depression by enhancing neurogenesis, increasing serotonergic neurotransmission, and modulating neuroinflammatory processes [20, 48, 51]. Additionally, the activation of AdipoR2 has been shown to facilitate the M2 polarisation of microglia, which in turn causes the secretion of neuroprotective factors and anti‐inflammatory cytokines [54]. Although we did not assess receptor expression levels directly, the existing evidence suggests that AdipoR1 is the primary mediator of the antidepressant effects of Adipo, with AdipoR2 offering additional support through its role in neuroinflammation and glial regulation. Further studies are needed to elucidate the specific functions of AdipoR1 and AdipoR2 in mediating the behavioural effects of Adipo, particularly in chronic SR.

Mel, a hormone that is primarily produced by the pineal gland in response to darkness, is essential for the control of wakefulness and sleep cycles and the circadian clock [55]. Mel has been demonstrated to enhance sleep quality and may alleviate specific depressive symptoms; however, its effects are generally moderate and differ significantly among individuals [56]. The increasing incidence of chronic sleep‐related conditions and their detrimental effects on psychological state highlights the need for innovative therapeutic strategies that go beyond Mel. In our study, Adipo was more effective than Mel at reducing anxiety and depression behaviours in sleep‐restricted mice. Notably, Adipo substantially decreased serum corticosterone levels, indicating that it effectively manages stress responses, whereas Mel did not elicit this effect. Furthermore, the administration of Adipo resulted in a marked decline in pro‐inflammatory markers and microglial activation, suggesting a neuroprotective mechanism. These results indicate that, in comparison to Mel, Adipo presents a more comprehensive and robust strategy for mood disorders associated with SR by addressing neuroinflammation and HPA axis dysregulation.

Although our results indicate that Adipo treatment in sleep‐restricted rodents is linked to a reduction in anxiety and depression‐related behaviours, as well as a lower level of neuroinflammatory markers, it is crucial to acknowledge a number of limitations. Initially, our molecular and histological analyses were confined to the PFC, and we did not evaluate other brain regions implicated in mood regulation, including the hippocampus or amygdala. This limitation suggests that Adipo may have effects that are either identical or different in these regions, necessitating additional research. Secondly, while we noted reductions in NF‐κB and IL‐1β expression in conjunction with behavioural changes, we did not employ pharmacological or genetic approaches to directly alter the NF‐κB pathway or other inflammatory mediators. Consequently, it is impossible to infer that the behavioural advantages of Adipo are exclusively derived from anti‐inflammatory mechanisms or NF‐κB inhibition. Future research should address these gaps in order to elucidate the mechanisms that underlie Adipo's effects in sleep‐restricted mice. Moreover, our use of a single dose of Adipo, based on prior dose–response findings, limited our ability to assess dose‐dependent effects or identify the minimal effective dose. It is recommended that future research investigate a broader range of concentrations to better define the therapeutic window and optimise treatment strategies. Additionally, this study exclusively employed male mice. Considering the impact of sex on the HPA axis and behavioural responses, subsequent research should incorporate both sexes to ascertain whether the effects of Adipo in chronic SR are uniform across genders. Finally, although we implemented standardised testing and handling protocols, the implementation of behavioural assessments on consecutive days may result in carryover effects. Future research should incorporate extended intervals between evaluations, counterbalanced test sequences, or distinct cohorts for various behavioural domains to address this potential confounding factor.

To further extend the translational implications of this research, subsequent investigations should incorporate neurophysiological approaches, such as EEG recordings, to evaluate the impact of Adipo on brain activity during chronic SR. Previous research has demonstrated that sleep deprivation is linked to sustained increases in NREM delta power, primarily in the frontal cortex, which are indicative of changes in neural function associated with sleep loss [57]. The incorporation of these neurophysiological parameters into future research will provide more profound insights into the effects of Adipo on brain function and behaviour in chronic SR.

## 5 Conclusion

In conclusion, our findings indicated that intranasal treatment of Adipo significantly alleviated anxiety, despair, and apathy‐like behaviours in sleep‐restricted mice by regulating the HPA axis, suppressing the NF‐κB pathway, and decreasing microglial activation in the PFC. This study suggests that Adipo may be a new therapeutic option for mood disorders linked to sleep problems, warranting further investigation into its mechanisms and effects in human populations.

## Author Contributions

**Beriwan Malaei:** methodology (equal), visualization (equal), writing – original draft (equal). **Fereshteh Farajdokht:** conceptualization (equal), formal analysis (equal), investigation (equal), methodology (equal), supervision (equal), writing – review and editing (equal). **Gisou Mohaddes:** conceptualization (equal), validation (equal), writing – review and editing (equal). **Behzad Mansouri:** methodology (equal). **Nasrin Hamidi:** methodology (equal). **Mohammad Reza Alipour:** conceptualization (equal), formal analysis (equal), funding acquisition (equal), supervision (equal), validation (equal).

## Conflicts of Interest

The authors declare no conflicts of interest.

## Data Availability

Data can be accessed upon reasonable request.
